# Stimulation of Cortical Myosin Phosphorylation by p114RhoGEF Drives Cell Migration and Tumor Cell Invasion

**DOI:** 10.1371/journal.pone.0050188

**Published:** 2012-11-19

**Authors:** Stephen J. Terry, Ahmed Elbediwy, Ceniz Zihni, Andrew R. Harris, Maryse Bailly, Guillaume T. Charras, Maria S. Balda, Karl Matter

**Affiliations:** 1 Department of Cell Biology, UCL Institute of Ophthalmology, University College London, London, United Kingdom; 2 London Centre for Nanotechnology, University College London, London, United Kingdom; 3 Department of Physics and Doctorate Program of Engineering of the Department of Chemistry, University College London, London, United Kingdom; 4 Department of Cell and Developmental Biology, University College London, London, United Kingdom; King's College London, United Kingdom

## Abstract

Actinomyosin activity is an important driver of cell locomotion and has been shown to promote collective cell migration of epithelial sheets as well as single cell migration and tumor cell invasion. However, the molecular mechanisms underlying activation of cortical myosin to stimulate single cell movement, and the relationship between the mechanisms that drive single cell locomotion and those that mediate collective cell migration of epithelial sheets are incompletely understood. Here, we demonstrate that p114RhoGEF, an activator of RhoA that associates with non-muscle myosin IIA, regulates collective cell migration of epithelial sheets and tumor cell invasion. Depletion of p114RhoGEF resulted in specific spatial inhibition of myosin activation at cell-cell contacts in migrating epithelial sheets and the cortex of migrating single cells, but only affected double and not single phosphorylation of myosin light chain. In agreement, overall elasticity and contractility of the cells, processes that rely on persistent and more constant forces, were not affected, suggesting that p114RhoGEF mediates process-specific myosin activation. Locomotion was p114RhoGEF-dependent on Matrigel, which favors more roundish cells and amoeboid-like actinomyosin-driven movement, but not on fibronectin, which stimulates flatter cells and lamellipodia-driven, mesenchymal-like migration. Accordingly, depletion of p114RhoGEF led to reduced RhoA, but increased Rac activity. Invasion of 3D matrices was p114RhoGEF-dependent under conditions that do not require metalloproteinase activity, supporting a role of p114RhoGEF in myosin-dependent, amoeboid-like locomotion. Our data demonstrate that p114RhoGEF drives cortical myosin activation by stimulating myosin light chain double phosphorylation and, thereby, collective cell migration of epithelial sheets and amoeboid-like motility of tumor cells.

## Introduction

Locomotion of single and groups of cells underlies dynamic biological processes ranging from development and tissue repair to tumor invasion and metastasis [1], [2], [3]. Actinomyosin contractility is an important determinant of cell migration during normal physiological and pathological processes. During tumor cell invasion and single cell migration, the importance of the actinomyosin cytoskeleton depends on the mode of migration. The forward movement of individual cells can be driven by actin-based, lamellipodial protrusions or actinomyosin contractility [3]. Actin-based protrusions drive migration of flat, mesenchymal-like cells; this mode of migration is hence often referred to as mesenchymal migration although it is also used by non-mesenchymal cells [1], [4]. Actinomyosin contractility is observed in roundish, amoeboid cells; hence, this mode of migration is generally referred to as amoeboid movement and is the primary mode of migration of highly motile cells such as neutrophils and some tumor cells. However, amoeboid movement is not a single mechanism, as the overall shape of the cells and motility are determined by the balance of adhesion, contractility and actin network expansion [5]. Tumor cells can employ either mechanism during invasion and can be forced to move in an amoeboid manner by inhibiting pericellular proteolysis [6]. Recent work with fibroblasts has also shown that, depending on the physical properties of the matrix, locomotion can be driven by lamellipodial extensions or actinomyosin contractility and that inhibition of myosin activity switches cells to lamellipodia-based migration [7].

During epithelial repair processes, models of collective cell migration, actinomyosin contractility along the leading edge is part of a purse string mechanism that leads to epithelial sheet closure [2]. Actinomyosin activity also occurs along cell-cell junctions close to wounds and is thought to be part of the mechanism that drives wound closure [8], [9]. However, whether cortical actinomyosin contractility at cell-cell junctions can promote collective cell migration is not known, as enhanced contractility upon inactivation of a negative regulatory mechanism leads to single cell migration [10].

Non-muscle myosin II, the main force-generating component of the actinomyosin cytoskeleton, is activated by phosphorylation of specific residues in its regulatory light chains (MLC) [11]. Serine-19 is the commonly phosphorylated site leading to myosin activation downstream of several signaling pathways including RhoA/ROCK and Cdc42/MRCK signaling [3], [12]. Additional Threonine-18 phosphorylation can also occur and, at least in vitro, enhances myosin activity at sub-saturating actin concentrations [13], [14]. However, the functional relevance of the double phosphorylation in intact cells is unclear. RhoA-stimulated myosin activation is critical for amoeboid movement and collective cell migration [3]. RhoA, as other RhoGTPases, is activated by guanine nucleotide exchange factors (GEFs) that are thought to control RhoA activation in space and time [15], [16]. Little is known about the RhoA GEFs that stimulate actinomyosin contractility during migration; however, identification of specific RhoA GEFs that drive tumor cell invasion would be important to design new therapeutic strategies to prevent tumor cell spreading and metastasis.

Here, we focus on p114RhoGEF, a RhoA activator that binds myosin IIA and regulates assembly of functional tight junctions [17], [18]. Our data now indicate that p114RhoGEF drives migration of epithelial sheets, and amoeboid movement and invasion of tumor cells. p114RhoGEF is not required for mesenchymal-like movement, which relies on Rac activation to push cells forward. p114RhoGEF activates cortical myosin by stimulating double phosphorylation of MLC along cell junctions close to leading edges and along the actin cortex of single cells. We propose that this unexpected mechanistic similarity between collective and single cell migration reflects a p114RhoGEF-activated myosin-dependent mechanism that drives cell shape changes and cortical actinomyosin dynamics required for locomotion.

## Materials and Methods

### RNA Interference

Human corneal epithelial (HCE) cells were generously provided by Dr. Min Chang (Verderbilt University, Nashville, Tennessee, USA) and were originally described by Araki-Sasaki et al., [19]. MDA-MB-231 cells were obtained from American Type Culture Collection. HCE and MDA-MB-231 cells were grown in high glucose DMEM containing 10% FCS as previously described [17], [20]. Cells were transfected with siRNAs using Interferin transfection reagent (Polyplus-transfection Inc.) [17], [21]. Non-targeting control siRNAs and siRNAs targeting RhoA, p114RhoGEF and GEF-H1 were obtained from Thermo Scientific (Dharmacon). All targeted sequences were as described previously [17]. In experiments in which individual siRNAs and pools of siRNAs were used, individual siRNAs are numbered and pools are labeled as ‘siRNA-p’. The total siRNA concentration was kept constant at 40 nM in all experiments.

### Immunological Techniques

Antibodies used were as follows: goat anti-p114RhoGEF (ARHGEF18), Everest Biotech; rabbit anti-myosin IIA, Sigma-Aldrich; mouse anti-Rock II, BD Biosciences; rabbit anti-MLC, mouse anti-p-MLC (S19), rabbit anti-pp-MLC (T18,S19) Cell signalling Technologies; mouse anti-Rac1-GTP, NewEast Biosciences; mouse anti-aPKCζ, Santa Cruz Biotechnology; mouse anti-α-tubulin [22]; rabbit anti-GEF-H1 [23]. Rabbit anti-p114RhoGEF antibodies were generated against a GST fusion protein containing the C-terminal domain [17]. The obtained sera were first run over a GST column and were then affinity purified using the same C-terminal fusion protein [24]. For immunoblotting of total cell extracts, cells were lysed directly in sample buffer and fractionated on 8.5% gels followed by immunoblotting as described [20]. Co-immunoprecipitations were performed as described [17]. For immunofluorescence, cells were either fixed in methanol (10 minutes at −20°C; stainings for p114RhoGEF, GEF-H1, α-tubulin) or paraformaldehyde (3%, 20 minutes) followed by Triton X-100 permeabilization (stainings for pp-MLC, p-MLC, myosin IIA, aPKCζ, fluorescent phalloidin). After blocking and incubation with primary antibodies, the samples were incubated with the appropriate fluorescent secondary antibodies conjugated to either FITC, Cy3 or Cy5 (Jackson ImmunoResearch Inc.). In some experiments, fluorescent phalloidin was used (TRITC, Sigma-Aldrich; Alexa647, Molecular Probes). Samples were processed and embedded as described [17], [25] and imaged using a Leica DMIRB fluorescent microscope using 63x immersion oil objective. Images were acquired using simple PCI and adjusted for brightness and contrast with Adobe Photoshop.

### Migration and Invasion Assays

HCE wound healing assays were performed by plating cells into two-chamber cell culture inserts in μ dishes (ibidi). Migration was then induced by removing the culture insert and followed by taking phase contrast images after the time points indicated using a Leica DMIRB inverted microscope equipped with a 10x objective. Images were acquired using simple PCI and open wound areas were calculated using ImageJ. For single cell migration assays, 48-well tissue culture plates were used that had been coated with the matrices indicated; 8000 cells were plated per well using HEPES-buffered medium (pH 7.4). Coating was performed at room temperature for 60 minutes using 100 µl of 100 µg/ml fibronectin (Sigma F1141), 100 µg/ml rat tail collagen I (Becton-Dickinson Labware), or 250 µg/ml Matrigel (growth factor reduced Becton-Dickinson Labware). For 3D assays, cells were plated on collagen I or Matrigel and, after attachment, the medium was removed and the cells were covered with 130 µl of 1 mg/ml collagen I or 2.5 mg/ml Matrigel, respectively. The matrices were left to gel for 2 hours at 37°C and then overlaid with tissue culture medium containing 10% fetal calf serum. After 16 hours, cells were transferred to a Zeiss Axiovert 200 M Microscope and time-lapse movies were recorded at 37°C for 5 to 12 hours using 5x or 10x objectives and simple PCI software. For quantification, cells were tracked with ImageJ and quantified with the Chemotaxis and Migration software tool (ibidi). Invasion assays were performed using polycarbonate filter inserts (6.5 mm diameter and pore size 8 µm; Becton-Dickinson Labware) similar to previously described protocols [26]. Filter inserts were pre-coated with 50 µl of 2.5 mg/ml Matrigel and left to polymerize for 2–3 hours at 37°C. After rehydration, 50,000 cells were plated inside the insert in media containing 1% fetal calf serum (FCS), and 10% fetal calf serum media was added outside the insert. Invasion of cells was evaluated every 24 hours by visual inspections of cells that migrated to the bottom of the well. After 72 hours of culture, the non-invasive cells were removed with cotton swabs and the inserts were fixed and stained with crystal violet. Pictures were taken, and invasive cells were quantified by extraction of crystal violet with acetic acid and determination of absorbance at 540 nm using a plate reader. Cells attached to the bottom of the dish were extracted with trypsin/EDTA solution and the cell numbers were determined using the CyQUANT assay (Invitrogen).

### RhoA and Rac Activation Assays

For RhoA and Rac activation assays, cells were transfected with control, p114RhoGEF and GEF-H1 siRNAs in 12-well plates. After 72-hours, cells were extracted and analyzed for levels of active RhoA and Rac using the respective G-LISA assay kit (Cytoskeleton Inc.) [17].

### Collagen Gel Contraction Assay

MDA-MB-231 cells were transfected with siRNAs in plastic dishes and were embedded in collagen 24 or 48 hours later. The collagen contraction assay was performed as previously described [27], [28]. Briefly, 24 (Experiment 1–3) and 48 (experiment 4,5) hours after transfection, MDA-MB-231 were trypsinised and embedded at a final concentration of 1.7 × 10^5^ cells/ml into a 1.5 mg/ml collagen matrix of rat tail collagen type I (First Link, UK) in 35 mm Mattek™ dishes, as previously described [28]. Following polymerisation, the gels were manually detached from the edges of the well and maintained in DMEM with 10% FCS. Gel contraction was recorded daily using digital photography and the gel area was measured using image J. Contraction is expressed as a percentage decrease compared to the original gel area. The result was not affected by the increased time between siRNA transfection and embedding in experiments 4 and 5.

### Cell Stiffness Measurements

To compare the stiffness of cells in different experimental conditions, we carried out AFM force spectroscopy measurements with a JPK Nanowizard-I (JPK instruments, Berlin, Germany) interfaced to an inverted optical microscope (IX-71, Olympus). AFM cantilevers with pyramidal tips (MLCT, Bruker, Karlsruhe, Germany) and nominal spring constants of 0.05 N.m^-1^ were modified by gluing 7.5 µm radius polystyrene beads (Invitrogen) to the cantilever underside with UV curing glue (UV curing, Loctite, UK). The spring constant of each cantilever was measured before affixing the bead using the thermal noise method implemented in the AFM software (JPK SPM). The sensitivity of the cantilever was set by measuring the slope of force-distance curves acquired on glass regions of the petri dish in which cells were plated (50 mm glass bottom Petri dishes, Willco wells, Amsterdam, The Netherlands). Prior to the experiment, the cell medium was replaced with Leibovitz L15 medium (Invitrogen) with 10% FBS. During AFM experiments, the tip of the cantilever was aligned over the centre of each cell and a force-distance curve acquired with an approach speed of 3 µm.s^-1^ and a target force of 1 nN. Stiffnesses were extracted from the force-distance curves by fitting the contact portion with a Hertzian contact model using custom-written Matlab routines as described in [29].

### Statistical Analysis

All graphs show averages and standard deviations. The corresponding n values are provided in the figure legends. The indicated p values were obtained with two-tailed Student’s t-tests.

## Results

### p114RhoGEF Drives Collective Cell Migration

**Figure 1:**
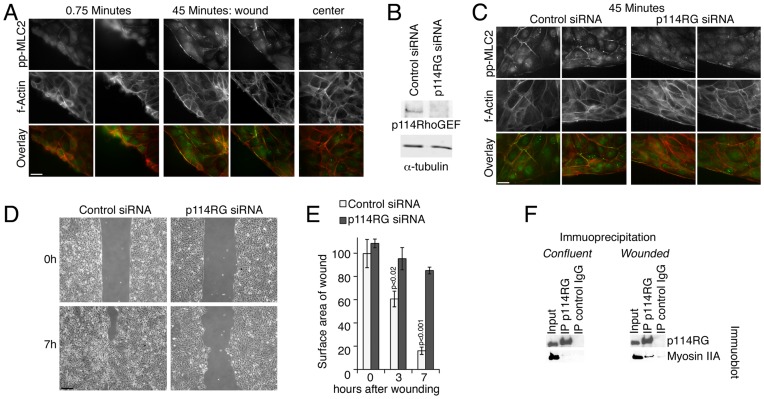
p114RhoGEF regulates myosin activation and migration during wound repair. (A) HCE cells, grown to confluence in dishes with two chamber culture inserts, were induced to migrate by removing the insert. Cell were fixed after different periods of migration and stained for double phosphorylated MLC and f-actin. Bar, 10 µm. (B,C) HCE cells were transfected with siRNAs and depletion of p114RhoGEF (B) and MLC phosphorylation (C) were analyzed as indicated. Note, MLC phosphorylation is only reduced at cell junctions, not at the leading edge, upon p114RhoGEF depletion. (D,E) Collective migration of HCE cells transfected with the indicated siRNAs was analyzed by measuring wound closure after different periods of time. (F) HCE cells were grown to confluence and left to stabilize for several days. The monolayers were then either directly extracted, or wounded with multiple scratches using a needle and re-incubated for 45 minutes prior to extraction. p114RhoGEF was then immunoprecipitated, and the precipitates were then analyzed by immunoblotting for the GEF and myosin IIA. Bar, 250 µm (D). Panel E shows means ±1SD, n = 3.

The actinomyosin cytoskeleton is an important component of epithelial junctions, and myosin II activity drives junction assembly and function [30], [31], [32]. Double phosphorylation of MLC at position Thr18 and Ser19 is low in established, mature monolayers in steady state. Upon wounding of human corneal epithelial (HCE) monolayers, phosphorylation was still low if cells were fixed immediately after wounding, but subsequently upregulated at cell-cell junctions in cells close to the wound and along the prominent actin belt along the leading edge (Fig. 1A). Hence, we asked whether p114RhoGEF, an activator of RhoA that associates with and activates myosin during junction formation, is also required for MLC phosphorylation during wound repair [17].

Figure 1B shows that p114RhoGEF was efficiently depleted by transfecting previously characterized siRNAs [17]. Depletion resulted in a strong attenuation of the induction of double MLC phosphorylation at cell-cell contacts, but not along the leading edge (Fig. 1C). Co-immunoprecipitation indicated that wounding stimulated enhanced association of myosin IIA with p114RhoGEF (Fig. 1F). p114RhoGEF thus stimulates myosin activation during epithelial repair in a spatially controlled manner at cell-cell junctions.

We next performed migration assays to determine the functional importance of p114RhoGEF during epithelial wound repair. Cells transfected with control siRNAs closed wounds rapidly within 7 hours, reflecting the efficient migration behavior of corneal epithelial cells [33]. If p114RhoGEF had been depleted, the two opposing cell sheets moved slowly and a large gap between them remained even after 7 hours (Fig. 1D,E). p114RhoGEF is thus required for collective cell migration of HCE cells.

### Regulation of Single Tumor Cell Migration

**Figure 2:**
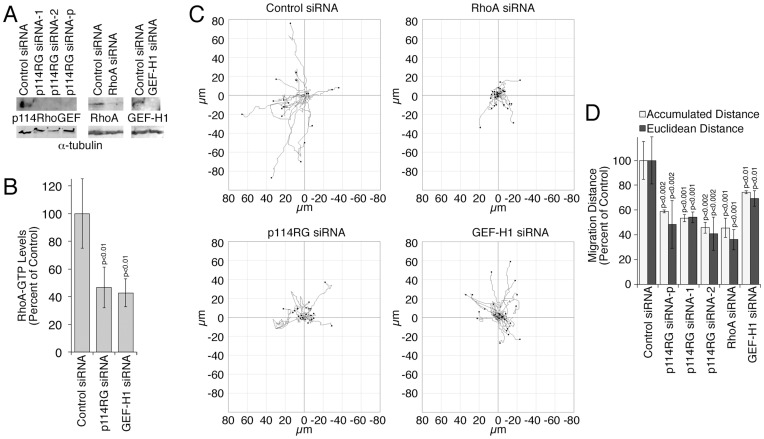
Regulation of single cell migration by p114RhoGEF. MDA-MB-231 cells were transfected with siRNAs as indicated (p114RG siRNA-1 and siRNA-2 refer to distinct individual siRNAs; siRNA-p refers to a pool of the two siRNAs). Expression of indicated proteins was analyzed by immunoblotting (A), effect on total levels of active RhoA by G-LISA assay (B; shown are means ± 1SD, n = 4), and migration by time-lapse microscopy over 5 hours (C,D). Migration distances were quantified by single cell tracking. Panel D shows means ± 1SD of four different fields (20 cells were analyzed for each field).

As p114RhoGEF regulates double MLC phosphorylation at cell-cell junctions, its role in cell locomotion might be limited to collective cell migration. To test this, we employed MDA-MB-231 cells, an invasive breast cancer cell line that expresses high levels of p114RhoGEF (Fig. S1). Transient transfections of siRNAs targeting p114RhoGEF led to efficient depletion of the protein in MDA-MB-231 cells (Fig. 2A). As positive controls, we depleted RhoA and an additional RhoA exchange factor, GEF-H1, known to stimulate RhoA close to the leading edge [34]. Depletion of both exchange factors, p114RhoGEF and GEF-H1, led to clear reductions in total RhoA activity (Fig. 2B). Time-lapse recordings followed by single cell tracking were then used to evaluate the importance of p114RhoGEF in single cell migration.

Figure 2C,D shows that depletion of RhoA and GEF-H1 inhibited migration as expected, confirming that locomotion of MDA-MB-231 cells is RhoA dependent. Transfection of siRNAs targeting p114RhoGEF also strongly attenuated migration (Fig. 2C,D; Movies S1, S2, S3). Hence, p114RhoGEF also regulates single cell motility. Depletion of p114RhoGEF resulted in a stronger inhibition than depletion of GEF-H1, despite a similar downregulation of total RhoA-GTP levels (Fig. 2B), suggesting that the two GEFs regulate different processes, as they do during epithelial differentiation [17].

Depletion of p114RhoGEF did not only lead to a reduction in migration but also to a change in cell shape, with cells becoming flatter and more spread (Fig. S2). Cells also moved in an apparently more mesenchymal-like manner forming pronounced lamellipodia at their leading edges (movies S1 and S2). Hence, p114RhoGEF might regulate locomotion of more roundish cells that tend to move in an amoeboid manner, but not of flat cells that migrate with the help of lamellipodial extensions. As mesenchymal-like migration is Rac-dependent, we next determined whether p114RhoGEF depletion led to a stimulation of Rac activation.

**Figure 3:**
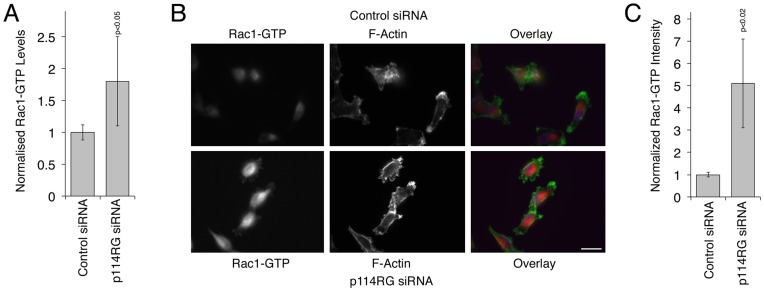
Rac activation in response to p114RhoGEF depletion in MDA-MB-231 cells. (A) MDA-MB-231 cells were transfected with the indicated siRNAs and the levels of active Rac were determined using a G-LISA assay 72 hours after transfection. Shown are means ± 1SD, n = 6. The numbers were normalized to control siRNA transfections. (B,C) Cells that had been transfected with siRNAs as in panel A were fixed and processed for immunofluorescence using an antibody specific for active Rac1 and fluorescent phalloidin. The intensity of the active Rac1 staining was quantified using image J. Panel C shows means ± 1SD of three experiments (in each experiment, at least 5 fields were analyzed).

Measurements of the cellular levels of active Rac indeed supported the conclusion that depletion of the RhoA activator p114RhoGEF led to enhanced Rac signaling (Fig. 3A). Similarly, staining for active Rac using an antibody specific for the GTP-bound form also indicated enhanced Rac activity in the cytoplasm as well as at the cell cortex (Fig. 3B,C). This thus indicates that depletion of p114RhoGEF expression indeed stimulated increased Rac activity.

Our results indicated that p114RhoGEF depletion led to flatter cells with more Rac activity, suggesting that the cells migrated in a more mesenchymal manner in its absence. Therefore, we next plated cells on different substrates to favor changes in migration modes to test whether there was a differential requirement of p114RhoGEF for efficient migration [5], [7].

**Figure 4:**
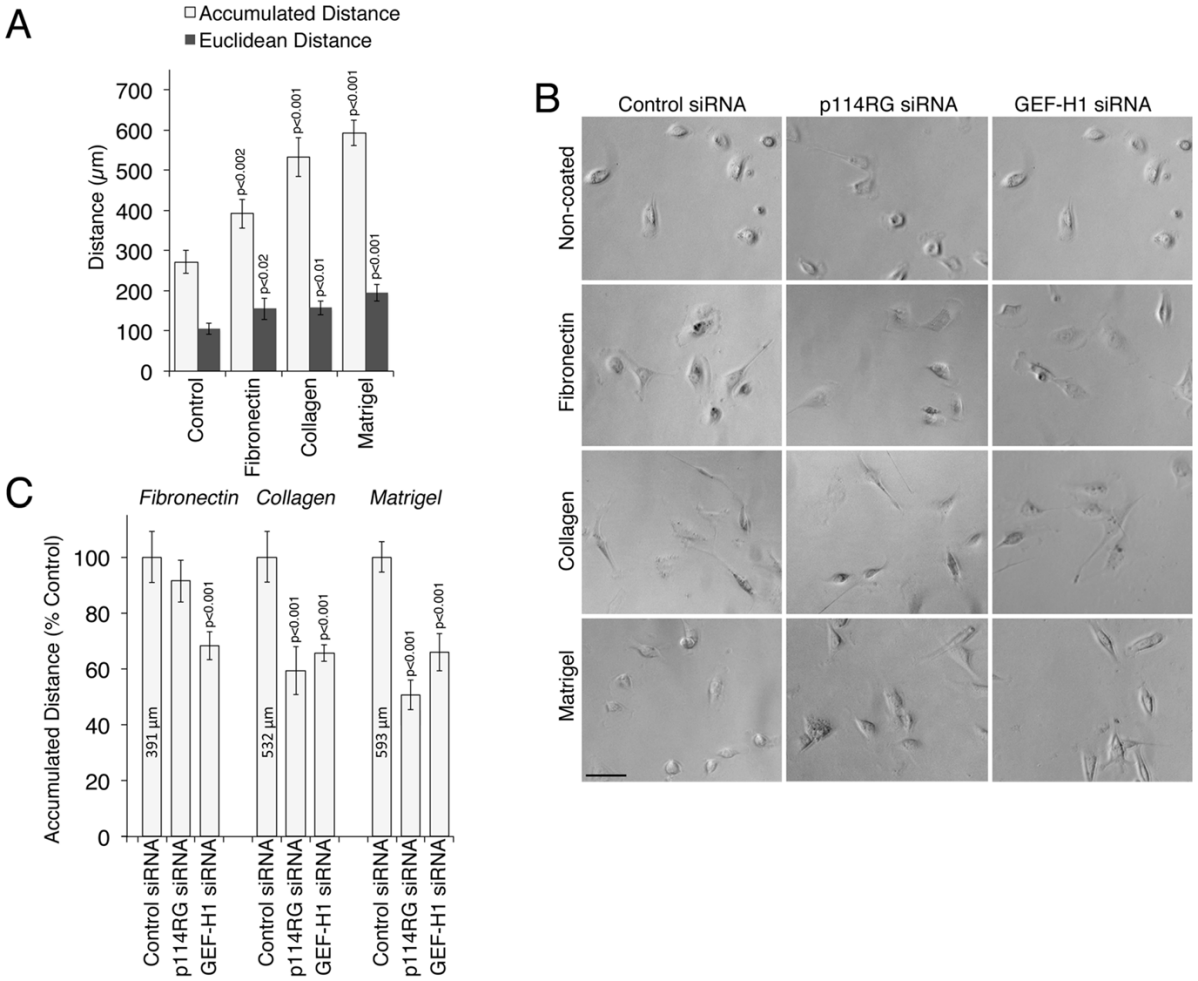
Matrix-dependence of p114RhoGEF-regulated migration. MDA-MB-231 cells were plated on the indicated 2D matrices and migration was analyzed by time-lapse microscopy as in figure 2 for 5 hours. In panels B and C, the cells had been transfected with the indicated siRNAs. All quantifications show means ± 1SD of four different fields (12 cells were tracked for each field). Note, only migration on collagen and Matrigel is p114RhoGEF-dependent. Bar, 30 µm.

Figure 4A shows that coating with extracellular matrix accelerated migration as expected [35]. Coating also affected cell morphology with cells on fibronectin appearing flatter and cells on Matrigel rounder (Fig. 4B). Collagen I coating stimulated roundish cell bodies but also stimulated long extensions. Knockdown of p114RhoGEF had a variable effect on the morphology of cells on coated surfaces and led to more spread cells on Matrigel, but the effect was not as pronounced as on non-coated dishes (Fig. 4B). Strikingly, cells on fibronectin were not only flat but also formed leading edges with pronounced lamellipodia; on Matrigel, cells moved preferentially as roundish cells.

We next quantified the effect of depletion of p114RhoGEF and GEF-H1 on migration of cells plated on different matrixes. Cells were transfected in large cultures on uncoated surfaces and then plated on the different matrices to ensure equal depletion efficiencies. Figure 4C shows that depletion of p114RhoGEF did not affect migration on fibronectin, whereas GEF-H1 depletion still attenuated it. On collagen and Matrigel, however, p114RhoGEF depletion inhibited migration effectively and was strongest when cells were plated on the latter substrate on which cells had the roundest morphology. GEF-H1 depletion inhibited migration on all matrices. p114RhoGEF thus regulates cell locomotion in a matrix-dependent manner and seems to affect amoeboid and not mesenchymal-like migration.

### Regulation of Tumor Cell Locomotion in 3D Matrices

Invasion of tumor cells into 3D matrices occurs either by mesenchymal- or amoeboid-like locomotion, with the latter being independent of MMPs [3], [6]. Given the phenotypes observed upon p114RhoGEF depletion in 2D migration, one would thus expect that invasion is p114RhoGEF dependent and that inhibition of MMPs does not affect p114RhoGEF-dependent invasion. To test this we performed first standard invasion assays across matrix coated filter inserts [1], [26].

**Figure 5:**
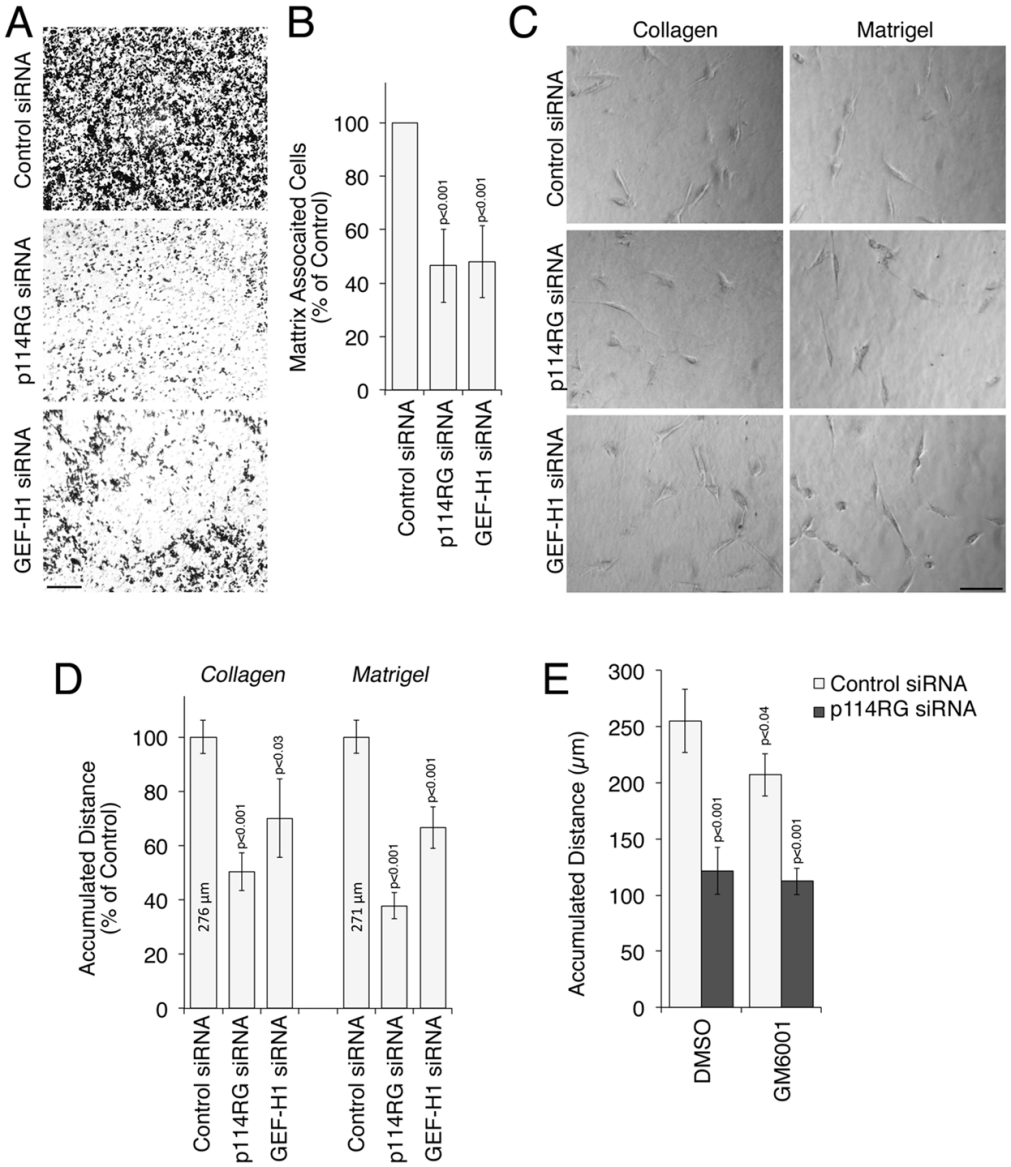
Regulation of tumor cell invasion by p114RhoGEF. (A,B) MDA-MB-231 cells were transfected as indicated and invasion across Matrigel covered filter inserts was then analyzed. Panel A shows images of the matrix after crystal violet staining. Panel B shows quantifications of matrix associated cells (shown are means ± 1SD, n = 7). (C-E) Cells transfected as indicated were plated into 3D matrices and migration as analyzed by time-lapse microscopy for 5 hours. Panel C shows still images and panel D quantification of migration distances of cells plated in collagen or Matrigel, and panel E migration in the presence of the metalloproteinase inhibitor GM6001. Quantifications show means ± 1SD of four different fields (12 cells were analyzed for each field).

Figure 5A shows that MDA-MB-231 cells efficiently invaded Matrigel coated filters. If p114RhoGEF had been depleted, however, invasion was drastically inhibited. Depletion of GEF-H1 also reduced invasion as expected [36], [37]. Quantification revealed that depletion of p114RhoGEF indeed inhibited invasion by about 60 percent (Fig. 5B). p114RhoGEF is thus required for efficient invasion.

We next embedded siRNA transfected cells into 3D matrices and recorded time-lapse movies to follow the movement and to quantify locomotion within the matrices. Figure 5C shows that the effect on cell morphology was not as striking as in 2D cultures. However, control siRNA transfected cells had again more roundish and cylindrical cell bodies and formed comparatively short extensions, typical for various forms of amoeboid-like, actinomyosin-driven movement [5]. Upon p114RhoGEF depletion, however, cells again appeared flatter and possessed longer extensions. Quantification of time-lapse recordings revealed that cells moved much more slowly through the gels if p114RhoGEF had been depleted (Fig. 5D, Movies S4, S5, S6). Depletion of GEF-H1 again revealed no clear effect on cell morphology and a more modest inhibition of locomotion.

To test whether inhibition of MMPs affects p114RhoGEF-dependent invasion, we repeated the assays in the presence of GM6001. The MMP inhibitor had only a modest effect on locomotion of MDA-MB-231 cells in Matrigel. If p114RhoGEF had been depleted, cells moved more than 50% slower than the respective control siRNA transfected cells. Hence, p114RhoGEF-dependent locomotion in a 3D Matrigel matrix is not MMP-dependent. This indicates that p114RhoGEF drives amoeboid movement during invasion.

### p114RhoGEF Stimulates Cortical Myosin Activation in Tumor Cells

**Figure 6:**
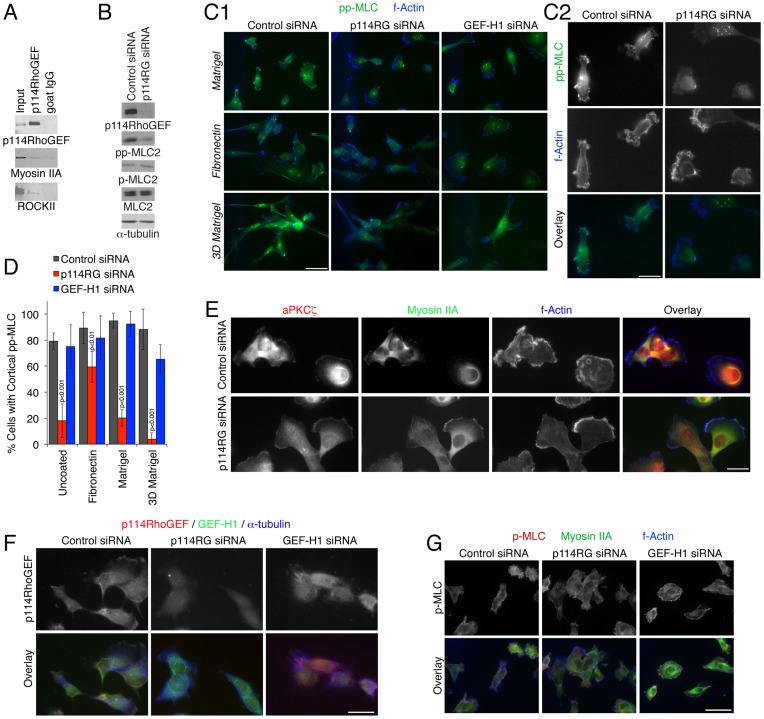
Regulation of cortical myosin phosphorylation by p114RhoGEF. (A) MDA-MB-231 cells were extracted and p114RhoGEF was immunoprecipated. The precipitates were then analyzed by immunoblotting for the GEF, myosin IIA, and ROCKII. (B) MDA-MB-231 cells that had transfected with the siRNAs indicated were lyzed and subjected to immunoblotting using the indicated antibodies. (C-G). Cells, transfected and plated as indicated, were fixed and then processed for immunofluorescence microscopy using antibodies against the proteins indicated. Panel C2 shows a larger magnification of the control and p114RhoGEF siRNA transfected cells plated on Matrigel shown in panel C1. Panel D shows a quantification of the percentage of cells with cortical staining for double phosphorylated myosin (shown are means ± 1SD). (C1, G) Bars, 10 µm; (C2, E, F) Bars, 5 µm.

During junction formation and collective cell migration, p114RhoGEF forms a complex with myosin IIA and stimulates its junctional activation as visualized by staining for double phosphorylated MLC (Fig. 1) [17]. Hence, we tested whether p114RhoGEF associates with and stimulates myosin in single cells. Figure 5A demonstrates that p114RhoGEF, myosin IIA, and ROCKII indeed co-immunoprecipitated from MDA-MB-231 cells, indicating that they form a complex in migrating single cells and, hence, that the Rho GEF is likely to stimulate myosin activation. This was further supported by immunoblotting results that indicated that p114RhoGEF depletion led to a strong reduction in double phosphorylation of MLC (Fig. 6B). Single phosphorylation of MLC was not detectably affected, suggesting that p114RhoGEF stimulates specifically double phosphorylation of MLC.

We next stained MDA-MB-231 cells with antibodies specific for double phosphorylated MLC and fluorescent phalloidin. Figure 6C shows that the antibody stained the cell cortex strongly along with some perinuclear staining. Cortical staining was observed on all matrices including Matrigel, fibronectin as well as 3D Matrigel. If p114RhoGEF was depleted, the strong cortical staining disappeared almost completely whereas the perinuclear staining remained at a similar level as in control cells. GEF-H1 depletion did not have clear effects on double MLC phosphorylation. Quantification of such images confirmed that p114RhoGEF depletion led to a strong reduction of cells with cortical double phosphorylated MLC in cells on all matrices except fibronectin, on which the effect was only small and migration was not p114RhoGEF dependent (Fig. 4C and 6D). p114RhoGEF thus stimulates cortical myosin activation in single cells in a matrix-dependent manner.

The f-actin staining further confirmed the morphological change of the cells upon p114RhoGEF depletion and the cells started to form the typical leading edge structures of cells during mesenchymal-like movement on 2D matrices (Fig. 6C). This was further confirmed by staining for atypical Protein Kinase C and myosin IIA, which accumulated at leading edges in a more polarized manner in p114RhoGEF depleted cells (Fig. 6E). p114RhoGEF itself localized to the cell cortex in a patchy manner, reminiscent of the double phospho-MLC staining. Similar to polarized epithelial cells, a considerable fraction of p114RhoGEF was cytosolic, and both cortical and cytosolic staining disappeared when p114RhoGEF was depleted (Fig. 6F).

The strong reduction in cortical double phosphorylation of MLC was surprising as p114RhoGEF depleted cells were still able to move, albeit more slowly. As the single phosphorylated form was not affected by immunoblotting (Fig. 6B), we also stained the cells for phospho-MLC (S19), which is less active at suboptimal actin concentrations in vitro [14]. Strikingly, depletion of p114RhoGEF did not detectably affect the staining, indicating that p114RhoGEF preferentially stimulates double phosphorylation of MLC (Fig. 6G).

**Figure 7:**
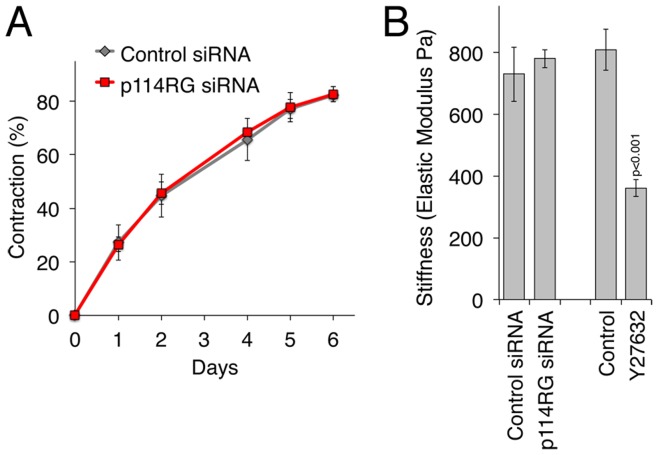
Analysis of collagen gel contraction and cell stiffness. (A) MDA-MB-231 cells were transfected with control or p114RhoGEF-specific siRNAs and were then embedded into collagen gels. Contraction of the gels was then followed for 6 days. Gel contraction was recorded daily using digital photography and the gel area was measured using image J. Contraction is expressed as a percentage decrease compared to the original gel area. Shown are means ± 1SD of 5 experiments. (B) Stiffness of MDA-MB-231 cells was measured by atomic force microscopy 72 hours after siRNA transfection. As a positive control, cells were incubated for 30 minutes with the ROCK inhibitor Y27632 prior to the start of the measurements. Shown are means ± 1SD of 3 experiments; in each experiment at least 30 cells were analyzed per condition.

Given the strong effect on double phosphorylation of MLC of p114RhoGEF depletion, it could be that this Rho activator stimulates overall contractility of the cells. Therefore, we measured the contraction of collagen gels by embedded MDA-MB-231 over 6 days. Although the cells contracted the gels efficiently, there was no difference between control and depleted cells (Fig. 7A). Hence, p114RhoGEF is not required for the persistent forces that stimulate gel contraction, suggesting that this process is primarily powered by single phosphorylated myosin.

As the reduced double phosphorylation of MLC may lead to reduced cortical stiffness, we also assessed the overall stiffness of cells using atomic force microscopy [29]. Figure 7B shows that cells transfected with control and p114RhoGEF-targeting siRNAs exhibited the same overall stiffness. In contrast, the measured stiffness was strongly reduced by ROCK inhibition, it therefore seems that also overall cell stiffness is primarily determined by the single phosphorylated form of MLC.

These data thus indicate that p114RhoGEF stimulates double, and not single, phosphorylation of MLC and that this form of active myosin is not required for parameters that rely on persistent forces, such as cell stiffness and gel contraction, but for more dynamic processes, such as cell motility.

## Discussion

Our data indicate that p114RhoGEF is a functionally important driver of cortical myosin activation during collective cell migration of epithelial sheets, and amoeboid-like migration and invasion of tumor cells, revealing an unexpected mechanistic similarity between collective and amoeboid-like single cell migration. Suppression of actinomyosin contractility by DDR1, an E-cadherin interacting protein, has recently been demonstrated to be required for the maintenance of cell-cell contacts during collective migration [10], [38]. Together with our data this indicates that collective cell migration requires careful balancing of inactivation and activation of actinomyosin contractility at cell-cell junctions. This is similar to the role of actinomyosin contractility during the formation and maintenance of cell-cell junctions in stationary epithelia as both junction formation and dissociation are regulated by RhoA/ROCK signaling [17], [32], [39], [40]. Thus, balancing of cortical actinomyosin contractility underpins cell-cell adhesion and dynamics in stationary as well as migrating epithelial sheets.

p114RhoGEF stimulates cortical myosin activation during amoeboid migration and invasion of tumor cells. Actinomyosin contractility is essential for amoeboid migration and RhoA signaling is an important driver of myosin activation during amoeboid migration [3]. To our knowledge, however, RhoA GEFs activating cortical myosin during amoeboid migration had thus far not been identified. Interplay between contractility and actin network expansion drive amoeboid migration of different cell types, leading to different cell shapes that generally share roundish cell bodies [5]; depletion of p114RhoGEF has thus led to cell flattening. As p114RhoGEF is widely expressed, it might drive locomotion of tumor cells from different tissues and not only from mammary epithelia.

It was surprising that a mechanism that stimulates junction formation is also required for amoeboid migration. However, as cells in a sheet move forward, junctions need constant remodeling to adapt cell shape changes and Rho-activated myosin activity is essential for junction dynamics [32], [39]; hence, myosin activity is required at cell-cell contacts during migration of sheets with intact cell junctions as it is during intercalation [41]. During amoeboid migration, cortical actinomyosin contractility provides actual force for forward movement [5]. Whether it does so also during wound repair or just drives junction remodeling to allow forward movement is currently unclear. However, it is possible that lateral actinomyosin driven contraction results in forward movement as long as adhesion sites more proximal to the wound edge provide the necessary traction.

The presence of p114RhoGEF also affected cell morphology of single tumor cells, with cells becoming generally flatter when it was depleted. However, the effect depended on the type of matrices and whether the cells were in a 2D culture. On fibronectin, for example, control cells were already flatter and p114RhoGEF depletion did not have a clear effect; whereas on Matrigel, control cells were more rounded but depletion of the GEF did not have such a strong effect as on uncoated dishes. This indicates that additional mechanisms contribute to cell shape determination and that the activity of such mechanisms is substrate-dependent. Junction-forming columnar epithelial cells such as the intestinal epithelial cell line Caco-2 also become flatter when p114RhoGEF is depleted and do not form tight junctions normally [17]. The data here now indicate that p114RhoGEF contributes to a more rounding or apically extended cell shape in a manner that is not directly dependent on cell-cell contacts.

MLC phosphorylation is a central mechanism of myosin activation [11]. If stimulated, p114RhoGEF forms a stable complex with myosin IIA and ROCKII that can be isolated from migrating cells and during junction formation (Fig. 1 and 6) [17]. Complex formation may favor double phosphorylation of MLC, causing the observed preferential effect of p114RhoGEF depletion on MLC phosphorylation. Although single and double phosphorylation at Serine-19 and Threonine-18 is well-established, the biological relevance of double versus single phosphorylation is not clear. As double phosphorylation leads to myosin that has a fully active ATPase at suboptimal actin concentrations *in vitro* [14], p114RhoGEF-induced double phosphorylation may favor cortical contraction even at cortex regions that have low f-actin concentrations, which would support overall cell rounding of single cells. The junctional cortex is rich in actin and, therefore, there may be additional consequences of double phosphorylation on cellular myosin activity that remain to be discovered.

p114RhoGEF depletion only attenuated cell motility but not overall contractility, as measured by collagen gel contraction, and cell stiffness. As these latter parameters require more persistent and constant forces than motility, it seems that p114RhoGEF functions in more dynamic processes such as cell migration or, as we have previously shown, tight junction formation [17].

Our data thus demonstrate that p114RhoGEF is a functionally important regulator of cortical myosin activity, driving migration of epithelial sheets, and tumor cell locomotion and invasion. These observations disclose an unanticipated mechanistic similarity between collective cell migration during wound repair and amoeboid motility of tumor cells. Our results further suggest that it might be possible to prevent tumor cells spreading and metastasis by designing approaches to block p114RhoGEF as part of new therapeutic strategies.

## Supporting Information

Figure S1 **Expression of p114RhoGEF in different epithelial cell lines.** Confluent cultures of the indicated cell lines were lysed and expression of p114RhoGEF was analyzed in total cell extracts by immunoblotting.
(TIF)

Figure S2 **Cell morphology of siRNA transfected cells.** MDA-MB-231 cells, transfected with siRNAs as indicated, were plated on uncoated dishes and time-lapse videos were recorded. Shown are still images illustrating the effect on cell morphology of p114RhoGEF depletion. Bar, 30 µm.
(TIF)

Movie S1 **Migration of control siRNA transfected MDA-MB-231 cells on non-coated dishes: 6 hours (100 frames); 10x objective.**
(MOV)

Movie S2 **Migration of p114RhoGEF siRNA transfected MDA-MB-231 cells on non-coated dishes: 6 hours (100 frames); 10x objective.**
(MOV)

Movie S3 **Migration of GEF-H1 siRNA transfected MDA-MB-231 cells on non-coated dishes: 6 hours (100 frames); 10x objective.**
(MOV)

Movie S4 **Migration of control siRNA transfected MDA-MB-231 cells in 3D Matrigel matrix: 12 hours (79 frames); 5x objective.**
(MOV)

Movie S5 **Migration of p114RhoGEF siRNA transfected MDA-MB-231 cells in 3D Matrigel matrix: 12 hours (79 frames); 5x objective.**
(MOV)

Movie S6 **Migration of GEF-H1 siRNA transfected MDA-MB-231 cells in 3D Matrigel matrix: 12 hours (79 frames); 5x objective.**
(MOV)
