# Supplementary figures and images for: Stimulation of Cortical Myosin Phosphorylation by p114RhoGEF Drives Cell Migration and Tumor Cell Invasion
Source: PLoS One. 2012 Nov 19;7(11):e50188. doi: 10.1371/journal.pone.0050188 (PMC3501466; doi:10.1371/journal.pone.0050188)

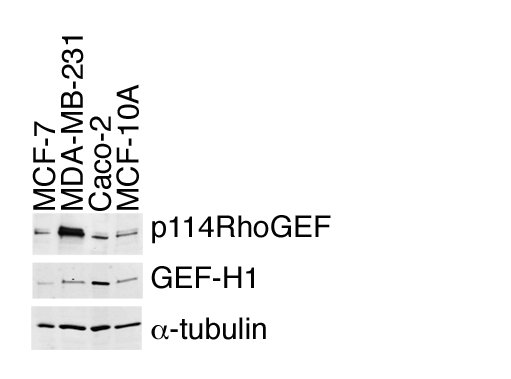

Supplement: Figure S1 — Expression of p114RhoGEF in different epithelial cell lines. Confluent cultures of the indicated cell lines were lysed and expression of p114RhoGEF was analyzed in total cell extracts by immunoblotting. (TIF) [file pone.0050188.s001.tif]

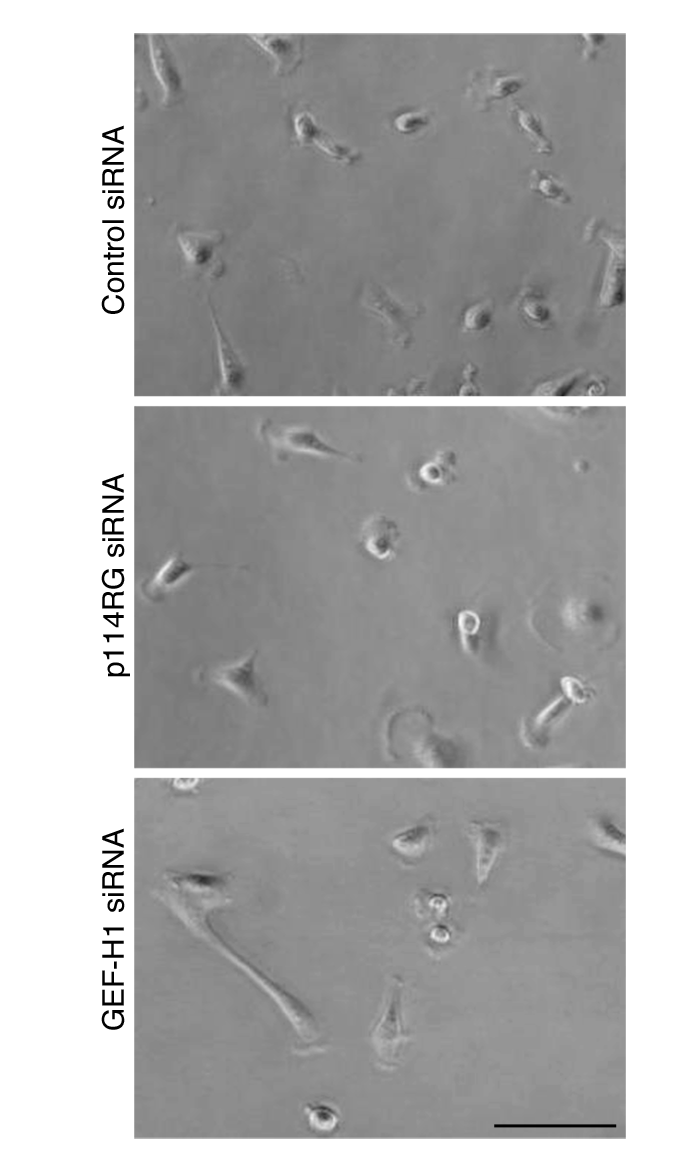

Supplement: Figure S2 — Cell morphology of siRNA transfected cells. MDA-MB-231 cells, transfected with siRNAs as indicated, were plated on uncoated dishes and time-lapse videos were recorded. Shown are still images illustrating the effect on cell morphology of p114RhoGEF depletion. Bar, 30 µm. (TIF) [file pone.0050188.s002.tif]
